# Identifying Facilitators, Barriers, and Potential Solutions of Adopting Exoskeletons and Exosuits in Construction Workplaces

**DOI:** 10.3390/s22249987

**Published:** 2022-12-18

**Authors:** Dilruba Mahmud, Sean T. Bennett, Zhenhua Zhu, Peter G. Adamczyk, Michael Wehner, Dharmaraj Veeramani, Fei Dai

**Affiliations:** 1 Wadsworth Department of Civil and Environmental Engineering, West Virginia University, Morgantown, WV 26506, USA; 2 Department of Mechanical Engineering, University of Wisconsin-Madison, Madison, WI 53706, USA; 3 Department of Civil and Environmental Engineering, University of Wisconsin-Madison, Madison, WI 53706, USA; 4 Department of Industrial and Systems Engineering, University of Wisconsin-Madison, Madison, WI 53706, USA

**Keywords:** Delphi, exoskeletons, exosuits, wearable devices, construction

## Abstract

Exoskeletons and exosuits (collectively termed EXOs) have the potential to reduce the risk of work-related musculoskeletal disorders (WMSDs) by protecting workers from exertion and muscle fatigue due to physically demanding, repetitive, and prolonged work in construction workplaces. However, the use of EXOs in construction is in its infancy, and much of the knowledge required to drive the acceptance, adoption, and application of this technology is still lacking. The objective of this research is to identify the facilitators, barriers, and corresponding solutions to foster the adoption of EXOs in construction workplaces through a sequential, multistage Delphi approach. Eighteen experts from academia, industry, and government gathered in a workshop to provide insights and exchange opinions regarding facilitators, barriers, and potential solutions from a holistic perspective with respect to business, technology, organization, policy/regulation, ergonomics/safety, and end users (construction-trade professionals). Consensus was reached regarding all these perspectives, including top barriers and potential solution strategies. The outcomes of this study will help the community gain a comprehensive understanding of the potential for EXO use in the construction industry, which may enable the development of a viable roadmap for the evolution of EXO technology and the future of EXO-enabled workers and work in construction workplaces.

## 1. Introduction

The construction industry is labor-intensive, physically demanding, and suffers from stagnant productivity and a high incidence rate of work-related musculoskeletal disorders (WMSDs). Occupational exoskeletons and exosuits (collectively called “EXOs” in this paper) are assistive devices that can support and reduce the physical load on workers performing demanding tasks [1,2]. Exoskeletons are mechanical devices that act as frames with motorized joints, whereas exosuits are flexible, soft, and lightweight [3]. EXOs have the potential to augment workers’ physical abilities, leading to improved productivity, reducing fatigue while performing repetitive tasks, and ultimately reducing the risk of WMSDs. The adoption of EXOs may also alleviate the situation of worker shortage that the construction industry is currently suffering from, as EXOs may enable workers to work more safely and for longer, help aged or physically incapable workers to maintain their employment for a more extended period, and attract a larger, more diverse pool of workers. However, despite these potential benefits, the adoption of EXO technology in the construction industry is still deemed challenging and lags behind that in other industries.

This paper aims to identify the potential facilitators and barriers to adopting EXOs in the construction industry, along with potential solutions to these identified barriers. To this end, the authors performed a multi-phase Delphi study through the organization of a workshop in which experts from academia, industry, and a government agency gathered, provided insights, and reached a consensus. The outcomes of this study will help to understand the status quo and the potential use of EXOs in the construction industry and shed light on their future development.

## 2. Background

### 2.1. Functions, Capabilities, and Potential of EXOs

An exoskeleton or exosuit is a wearable technology that can minimize tension in and injury to different parts of the body of a person by providing joint support, distributing weight, and correcting posture [4]. The design of occupational EXOs is aimed at the smooth interaction of the devices with their users, considering the whole workspace, by improving the safety and comfort of the user and optimizing overall system performance without obstructing natural kinematics or resulting in any discomfort or injury [5]. EXOs can be classified into different categories based on different characteristics. Based on the nature of the assistance and power source, they can be categorized into active, passive, and semi-active EXOs. Active EXOs are equipped with power sources, such as electric motors, hydraulic actuators, pneumatic muscles, or other sources of stored energy, to enable the user to move faster or lift or carry heavier loads by providing additional energy to the system [6]. Passive EXOs store energy through springs, dampers, or other materials generated by the user’s movement and then utilize that energy to augment power in other body parts that require support [1,6]. The third type of EXOs are termed semi-active EXOs [7,8] or quasi-passive EXOs [9], for which low-power actuation units are used to modify the spring-based actuation mechanisms of passive devices so as to enable passive exoskeletons with a certain degree of adaptivity. Based on the supported body parts, EXOs can be classified into upper-extremity (arms, shoulders, and upper torso), lower-extremity (legs, hips, and lower torso), and full-body (both upper and lower torso) EXOs [6]. Another classification is based on the robotic mechanisms of EXOs, with EXOs being classified into three types: anthropomorphic, quasi-anthropomorphic, and non-anthropomorphic [10]. Anthropomorphic EXOs are designed by aligning the rotation axis of the robot joint with the human joint, which enables it to mimic the user’s motions. Quasi-anthropomorphic EXOs have joints functionally like human joints but not aligned with the rotation axis of the human joint. Non-anthropomorphic EXOs have the robot joint designed in misalignment with the human joint [10].

EXOs enable users to move faster, carry heavier loads, and perform repetitive tasks with greater endurance. These EXO-enabled capabilities can be leveraged in the construction industry for increased productivity and safer construction workplaces. Various types of EXOs may be employed in construction workplaces [4]. Power gloves and handling EXOs help in lifting and holding heavy objects and tools for long durations. Arm and shoulder EXOs support those body parts in lifting, holding, and repetitive-arm-movement activities, such as cutting, drilling, and scraping, by reducing strain during job performance. Back-support EXOs play a vital role in reducing lower back disorders while lifting heavy objects and working in a forward-leaning posture, diminishing the stresses on back muscles. Leg-support EXOs enable users to maintain prolonged standing and crouching postures, which are common in various construction tasks, and reduce the stress on knees and legs by transmitting the force directly to the ground [4]. A full-body EXO has the potential to turn a user into a super-worker with the capability of safely lifting and manipulating up to 200 pounds [2]. Non-anthropomorphic EXO joints are misaligned with human joints and provide more diverse functionality for more convenient motions and effective energy consumption in performing tasks [10].

### 2.2. State of Research on the Application of EXOs in Construction

The construction industry—a major contributor to the global economy—is slow to adopt automation. Nnaji et al. [11] conducted a survey among 102 construction practitioners to identify the benefits and limitations of using technologies in the construction industry for safety and health management and provided invaluable information regarding barriers to technology adoption and strategies to overcome these barriers. This study pointed out the barriers to technology adoption in the construction industry in general and provided valuable insights for understanding the barriers to adopting EXOs [11]. Kim et al. [12], on the other hand, carried out a qualitative content analysis through phone interviews with 26 representatives of the construction industry, including a vice-president of a company, a project manager/engineer, a safety and health manager/director, and carpenters, to gain their perspectives regarding the benefits of, the factors involved in, and the barriers to adopting EXOs. Zhu et al. [2] reviewed 85 research articles to synthesize insights regarding the potential for using EXOs in the construction industry. The authors also generated a map suggesting types of EXOs for different trades by evaluating the benefits and challenges [2]. Through an evidence mapping systematic review, De Bock et al. [9] provided an overview of the literature related to the assessment of occupational EXOs and proposed a literature-based framework for benchmarking the effects of future EXOs on users. In this study, they reviewed 139 articles in which the effects of one or more EXOs on users were summarized, including 33, 25, and 18 unique back, shoulder, and other EXOs, respectively. Okpala et al. [13] conducted a study of a multiphase process that included a detailed literature review, an online survey, and a lab-simulated usability study. In this study, the authors evaluated the suitability of 11 EXOs for the construction industry and determined that these EXOs could prevent about 60% of WMSDs and 30–40% of accidents. They also identified the barriers to acceptance of these EXOs [13].

Research has also been conducted to collect scientific data to examine the efficacy of EXOs. Kim et al. [14,15] carried out laboratory assessments in two consecutive studies to identify expected and unexpected effects of a passive EXO for arm elevation on discomfort, shoulder muscle activity, and task performance and observed that it reduced shoulder muscle activity and task completion time substantially. Antwi-Afari et al. [16] assessed a passive exoskeleton system on spinal biomechanics during repetitive manual material handling tasks with the help of surface electromyography (sEMG) in the laboratory and observed that the system reduced extensor moments and stresses in internal muscle and lumbar regions. The participants reported that perceived discomfort-level scores for the lower back regions were also reduced. Golabchi et al. [17] evaluated the rate of perceived exertion, level of discomfort, overall fit and comfort, effectiveness, and interference level while wearing EXOs adopting different postures during dynamic and static material handling tasks. Though the participants reported an elevated level of discomfort, especially on the chest, they rated EXOs for usability and effectiveness, and the authors concluded that EXOs have the potential to reduce MSDs in construction after proper training for appropriate postures. Gonsalves et al. [18] assessed a back support EXO in terms of task performance and physiological conditions while performing a rebar placement task and concluded that the EXO has the potential to reduce lower back disorder. The study indicated that the EXO improved productivity and decreased perceived discomfort, although the participants reported discomfort in the chest. Cho et al. [19] designed and developed an EXO that can effectively keep workers in safer postures while performing a task and be used as a safety training tool. The effectiveness of a postural-assist EXO was examined by Ogunseiju et al. [20] for manual material handling tasks and it was revealed that the participants were significantly responsive to the feedback from the EXO and rectified any unsafe postures.

## 3. Problem Statement and Research Objective

EXOs have long been used for medical, military, and manufacturing applications, but in the construction industry, the use of EXOs is in its infancy. While efforts in the extant literature have led to understanding of the benefits, limitations, challenges, and opportunities of EXO use in the construction industry, insights have primarily been sought only from professionals within the construction industry or by summarization of the existing literature. As the successful acceptance, adoption, and application of EXOs in the construction industry depends on multiple relevant stakeholders, such as EXO manufacturers, contractors, robotic and mechanical experts, psychologists, insurance companies, economic specialists, and government agencies, a holistic understanding of their collective inputs regarding the facilitators and barriers and their potential solutions is still lacking. Gaining a comprehensive and multi-faceted perspective from all the stakeholders is essential to developing a roadmap for EXO development and adoption in construction.

To fill this knowledge gap, the objective of this research was to identify the facilitators of and barriers and potential solutions to the adoption of EXOs in construction workplaces by acquiring convergent insights from multiple stakeholders through a sequential, multistage Delphi study. Experts from academia, industry, and a key government agency participated in this study and shared insights and reached a consensus with respect to the issues identified above.

## 4. Methodology

This research adopted a consensus development method known as Delphi, in which brainstorming and discussion among a group of experts familiar with the subject and with adequate knowledge to provide inputs for a better understanding of the facilitators and barriers and potential solutions to drive the adoption of EXOs in the construction industry took place. The Delphi method is designed to reach the most reliable consensus on the basis of opinions collected from a group of experts [21]. The detailed procedure of the Delphi method implemented in this research is presented below.

The first step was to select an expert panel that included individuals with relevant knowledge and experience. A survey questionnaire was prepared on Qualtrics to ask about the experts’ level of education, years of work experience, current position, level of familiarity with EXOs, and their point of interest in this new technology for implementation in the construction industry. The questionnaire was sent via email to potential experts to determine the relevance of their credentials in fields related to EXOs and their interests in EXO implementation in construction workplaces. Based on the responses received, 18 experts were invited to participate in a workshop where the Delphi method was applied in three rounds. In this process, six categories, namely, business, technology, organization, policy/regulation, ergonomics/safety, and end users (construction-trade professionals), were chosen for brainstorming and input. These categories were developed based on their relevance to EXO use in construction in terms of goals, outcomes, processes, and beneficiaries, as well as a review of the relevant literature on technology adoption [22,23,24] that formed a lens allowing a view of the barriers and enablers from a holistic perspective. 

It was essential to have at least one expert in each category so that all possible perspectives could be discussed and the ideas verified from a practical point of view. The expert panel comprised representatives from academia, industry, and government, as shown in Table 1.

Table 2 details the work experience of the experts and the percentages of attendees in each group of experts (academia, industry, and government agency).

**Figure 1:**
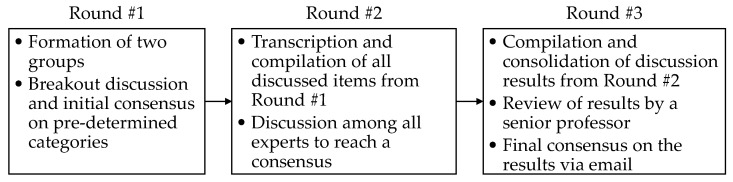
Schematic diagram of the three-phase Delphi process.

The workshop was held in Morgantown, WV, over one day. At the beginning of the workshop, the experts were presented with the preliminary findings of field experiments [25] conducted by the authors to bring them up to date regarding EXO studies in construction. The experts were also informed about the procedure and expected outcomes of this activity. Then, the Delphi process was initiated, as illustrated in Figure 1.

In the first round, the experts were divided into two groups with an equal number of participants for brainstorming and discussion. Each group discussed the facilitators and barriers and their potential solutions with respect to the six predetermined categories mentioned earlier. The authors endeavored to maintain group cohesion by equally distributing experts from each sector in academia, industry, and government and evenly separating experts in the same work area (e.g., EXO manufacturers). Each expert was provided with a tabulated handout containing spaces for providing written input on the facilitators, barriers, and potential solutions for each category. All the experts in each group shared their written opinions and evaluated others’ viewpoints. The authors adopted the “include reasons” method to minimize the effects of bias, which is a potential concern with the Delphi process [26]. The “include reasons” method required each expert to explain their reason for selecting each issue or item that they raised for discussion. Once completed, these responses were collected from each group and transcribed into a tabular format during a recess by four delegated graduate students. In the second round, the experts were presented with the tabulated results from the first round of discussions. Together, they were asked for their opinions and explanations, if necessary, to gain a better understanding of the collected discussion responses. During this round, disputed items were removed and new items were added to the group discussion in the tabulated form, resulting in a table that represented the complete consensus of the group. Additionally, among the identified barriers, those with high priority were also identified by group consensus. In the third round, following the workshop, the authors refined and consolidated the outcomes from the workshop. The outcomes were then reviewed by a senior professor to enhance the comprehensibility of the items following peer debriefing [27] and sent via email to all the experts for a final review and consensus. The finalized outcomes from this three-round Delphi process are discussed in detail in Section 5.

## 5. Outcomes of the Delphi Process

The expert panel discussed the facilitators, barriers, and potential solutions in six categories: business, technology, organization, policy/regulation, ergonomics/safety, and end users (construction-trade professionals). The following presents the discussion outcomes in detail.

### 5.1. Business

From a business standpoint, EXOs have to provide perceived job relevance and usefulness that allow for their incorporation into the process of a construction project. The business values that EXO use can bring about are vital for construction companies to make any adoption decision.

#### 5.1.1. Results

Table 3 summarizes the outcomes from the discussion of the expert panel regarding the facilitators, barriers, and potential solutions in the business category.

#### 5.1.2. Facilitators

EXOs are designed to limit the risk of injuries and increase workers’ productivity. Where a worker may need a companion to lift a heavy weight, by using an exosuit s/he alone may be able to lift the load. Therefore, fewer workers might be required to perform a given task and the other workers present can be utilized for different tasks, significantly increasing productivity. Moreover, wearing EXOs can be assistive in providing psychological support to workers and enable them to work more efficiently, as a worker may intuitively derive aspiration and inspiration from EXO use. Eventually, increased productivity yields higher profits for businesses, thereby driving higher adoption of EXOs in industry.

EXOs are new technologies in the construction industry. If EXOs are adopted in this industry, then construction firms will hire more professionals to monitor and maintain the products and keep them in good working condition and train workers for effective EXO use. In addition, experts will be needed in the industry for the certification of the products. This will create more job opportunities in the construction industry. Since EXOs can reduce the risk of WMSDs among workers, this will lead to a reduction in worker compensation costs for construction companies. Moreover, the construction industry has been facing a shortage of skilled-trade workers for a long time. If workers can perform more tasks per time unit with the use of EXOs, the additional production per time unit will be a significant benefit and help businesses mitigate the challenges of worker availability.

#### 5.1.3. Barriers

The prices of EXO products can currently range from $5000 to $100,000 and hence are not affordable for workers to purchase for their use. The initial investment required to purchase EXOs can be prohibitive if a company wants to provide EXOs to all its workers. Moreover, there is an additional cost for ongoing maintenance of EXOs to ensure workers’ safety. Even if a company were to purchase EXOs for its workers, there is a possibility of workers being reluctant to and even rejecting the use of this new technology. Therefore, companies are hesitant about investing in EXOs.

In the business category, a prime barrier to EXO adoption is the lack of data providing validated evidence of the benefits that EXOs can offer to contractors and businesses. The use of EXOs is currently so limited in construction workplaces that there is a dearth of data to justify EXOs’ usefulness and benefits relative to financial and other risks. EXO manufacturers claim various benefits based on EXO uses in other industry sectors, but these may not be valid for the construction industry due to differences in the nature of the tasks that need to be performed and the requirements for construction relative to other industries. The lack of data and low usage of EXOs in the construction industry causes skepticism among construction companies regarding the potential benefits of EXOs.

As EXOs have not been widely used in the construction industry, the potential returns on investment (ROIs) and even how to estimate ROI are unknown. For this reason, the industry is hesitant about investing in EXOs without having a proven ROI. There needs to be a standard approach for linking benefits to the changes enabled by EXOs, allowing for the measurement and quantification of EXO impacts on injury prevention and/or production improvement and thus the resultant financial benefit to companies.

Currently, there are not enough professionals in the industry with the requisite knowledge and expertise in testing, implementing, and maintaining EXOs. This lack of skilled professionals is a barrier to the adoption of EXOs in the construction industry. As there are no clear assignments for the mandatory use of EXOs in construction companies, staff (contractors and engineers) are reluctant to spend time on trials as they are already swamped by their daily duties. In addition, workers need training in the efficient use of EXO products but there are not enough experts in the industry to provide the necessary training. Even when skilled training for EXO use is available, construction companies have to be willing to invest money in training their employees. Furthermore, if EXOs are adopted by construction companies, the products need to be adequately maintained to avoid accidents due to worn-out or faulty equipment. For proper maintenance, businesses will need staff employed for this duty with proper expertise and skills, which would further add to the cost of adopting EXOs. Finally, as EXOs are new technologies, and even more so in the construction industry, there need to be more experts who can certify the products and provide proper training, as well as healthcare personnel who can verify whether the use of a product is appropriate for a particular worker.

Currently, the range of EXO products on the market for construction companies is limited. This limited selection of EXOs further hinders the ability of construction companies to conduct trials and evaluate EXOs for adoption in construction workplaces.

Additionally, the impact of the use of EXOs on work-pattern shifts is unknown. As workers work in shifts, the work patterns for projects may change depending on who will use the EXOs, for which tasks, when, and for how long. Further, there is no established decision-making process to determine how EXOs will be assigned to workers, by whom, and based on which criteria.

#### 5.1.4. Potential Solutions to Barriers

As the lack of knowledge regarding EXOs among construction businesses and trade workers has been identified as a barrier to EXO adoption, one potential solution is for EXO manufacturers to offer training programs in partnership with trade schools, contractors, associations, and apprentice training programs to increase awareness and knowledge about EXOs and their use in industry. Campaigns can be conducted to inform workers, contractors, and relevant firms about the benefits and risks of using EXOs. As EXOs are designed to help workers with heavy work with ease and reduce the risk of injuries, the adoption of EXOs is expected to benefit construction workers, which is a primary aim of unions. As a result, unions may help promote the use of EXOs by advocating this technology and educating workers at various occasions, such as meetings and speeches by opinion leaders.

The more opportunities that workers have for using and practicing with EXOs, the more confident and efficient they can be in using them. Hence, it would be beneficial for workers if they were provided with opportunities to use EXO products outside of workplaces (e.g., home remodeling).

EXOs can be incorporated into high school and college curricula to build awareness among students before they join the workforce as professionals upon graduation. Providing access to EXOs to students will allow them to study, experiment with, and assess the technology, which will eventually help reduce the gaps in knowledge about EXO use and improve EXO products to make them more viable in construction. Moreover, colleges can provide EXO education to working professionals in the construction industry and thereby create more EXO experts and trainers.

The involvement of healthcare professionals (including doctors and physiotherapists) can aid in the propagation of information regarding the benefits of EXOs and thereby help in the adoption of EXOs. They can promote EXOs during various meetings with worker patients and informational campaigns sent to patients. Additionally, if the working capacity of a worker is compromised after an injury, then with the help of EXOs they may gain the ability to return to work, saving them from unemployment. Healthcare professionals can thereby assist this process by recommending EXOs to appropriate patients.

Construction businesses can grow their understanding of EXOs by working with EXO experts. Conducting trials with EXOs within companies can also be facilitated by these experts.

Additional scientific research on the use of EXOs in construction will help to collect more data regarding such factors as benefits, pains, injuries, discomforts, and soreness. The data and findings from such research efforts will not only help support the use of EXOs but also help EXO manufacturers in improving their products.

It will also be beneficial to conduct more real-world case studies to collect companies’ feedback regarding their experiences with EXOs. If companies participate in case studies, then data collection regarding the impacts of the use of EXOs will be quicker, relevant, and more credible.

#### 5.1.5. Mapping Solutions to Barriers

**Figure 2:**
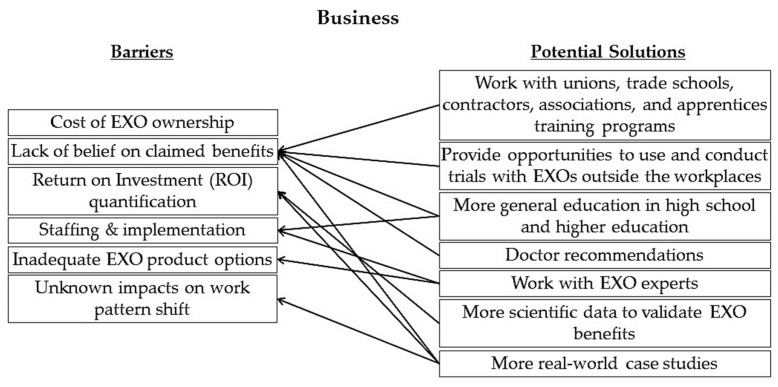
Mapping of potential solutions to identified barriers in the business category.

During the workshop, the expert panel identified barriers in the business category as well as potential solutions to those barriers. Figure 2 shows the mapping of the potential solutions to the identified barriers. No obvious solutions were identified for the barrier of “cost of EXO ownership”, calling for further studies to explore solutions to this issue.

### 5.2. Technology

From a technological standpoint, the level of readiness and the functional or operational maturity of the current EXO products play an important role in the decision-making process for adoption among the construction industry.

#### 5.2.1. Results

Table 4 depicts the compiled outcomes from the discussion of the expert panel regarding the facilitators, barriers, and potential solutions in the technology category.

#### 5.2.2. Facilitators

Venture capital and private equity firms in the technology investment sector can help drive the accelerated development of EXO technology and its adoption in various industries, including the construction sector.

State-of-the-art real-time feedback technologies, such as iWatch and other sensors, can be facilitators for the technological advancement of EXOs. While workers are wearing EXOs during work, these technologies can provide physiological measurements of the impacts of EXOs on the workers’ bodies and signal warnings if potential risks (e.g., high stress) are identified. Such real-time feedback technologies, in turn, may help workers feel more confident about using EXOs in construction workplaces.

#### 5.2.3. Barriers

As EXOs are new in the construction industry, research is needed on the performance of EXOs in construction applications. For construction companies, making investments in EXO research and development can be perceived as risky from an ROI perspective. Similarly, EXO manufacturers face the cost challenges of R&D as well, given the uncertainty of sustainable financial gains from investment in technological development for the construction industry.

EXOs can impose a variety of constraints on work performance in construction jobsites and these can hinder the adoption of this technology. Bulky size and limited movement may cause additional risks of accidents. The additional time needed for donning and doffing may reduce the amount of time during a work shift that a worker can be productive. To mitigate this negative impact on project productivity, construction workers who use EXOs will need to wear them for a relatively long duration, possibly the entire shift. Hence, it is essential for the EXOs to be lightweight, comfortable, easy to don and doff, and flexible enough not to constrain movement, which is not the case at present for commercially available EXOs.

Construction workers must wear personal protective equipment (PPE) at construction sites whenever necessary. For example, a safety harness is required for working at an elevated height. If workers wear EXOs together with PPE, then compatibility could be a concern. EXOs must not compromise the functional use of PPE that protects the safety and health of workers. In elevated settings, EXOs need to be incorporated with safety gears gracefully, so that the gears do not restrict a worker’s movements and cause imbalance, otherwise EXOs could be a source of additional injury risk for workers wearing PPE. Furthermore, most EXOs are task-oriented, which means that additional time is required to switch from one task to another. Finally, active EXOs tend to be heavy and bulky and harder to carry, making it challenging to integrate them with PPE.

Currently, there are no standard performance metrics to assess EXOs in terms of improved safety, increased productivity, reduced costs, and other factors. Testing procedures are also different for different types of EXOs and are dependent on the testing location and setup. This lack of standardization of performance metrics makes it difficult to assess and compare the performance of EXOs and consequently to justify investment in this technology.

EXO products may have built-in tracking units that track the equipment’s position, trajectory, as well as the user’s physiological responses and other private data. These data help to monitor the worker’s condition and oversee the project activity but can constitute confidential information about the worker. Therefore, there is a privacy concern for workers regarding how these data are to be used, shared, and protected.

#### 5.2.4. Potential Solutions to Barriers

Incremental improvements in EXO design and functionality that address the current challenges and limitations of the technology (such as weight and motion constraints) can help to increase EXO adoption in the construction industry. Such progressive upgrades can help to improve the time of adjustment when using these products and switching from one task to another, easing immediate use. Making ongoing incremental improvements to EXOs will require smaller amounts of investment capital, thereby reducing investment risks.

PPE, such as safety harnesses, is an integral part of a construction worker’s outfit. Therefore, it is essential for EXOs to perform seamlessly with PPE and other tools used by workers for their jobs. More investigations and assessments are needed to integrate PPE and tools with the use of EXOs in elevated settings.

Data regarding the impact of EXO use by workers, if collected in near real time with the help of wearable sensors, can provide essential feedback for the improvement of products and warn users regarding any safety issues associated with the use of EXOs. Additionally, such data will offer evidence of the benefits of EXO usage, leading to greater understanding and adoption of EXOs in the industry.

Task-oriented EXOs are designed to perform specific tasks. For instance, power gloves are intended for gripping tasks, while tool-handling EXOs are used for holding heavy tools, such as drilling equipment [4]. While these types of EXOs are less complex and efficient for performing a single task, switching between tasks takes time. Function-oriented EXOs are based on functionalities, such as preventing accidents, reducing fatigue, and increasing capability. Anatomy-oriented EXOs are classified based on which part of the body is supported by the EXO, such as upper- or lower-extremity EXOs. Among all these types of EXOs, it is essential to identify which types are most suitable for the construction jobsite depending on their effectiveness, efficiency, and comfort. For instance, if a worker is tasked with a drilling job, then task-oriented (tool-handling) EXOs are most suitable, whereas if the worker has to stand for an extended duration to perform their job, s/he needs support for the lower extremities and a hybrid or full-body EXO would seem more appropriate in this case.

Currently, there is no clear research-based evidence regarding which types of EXOs are most effective for use on construction sites. Task-oriented EXOs are suitable for specific tasks but limit workers to specific tasks, thus consuming more time in the donning and doffing of EXOs during the work shift. A full-body EXO may be more suitable for performing several tasks but it can present additional movement constraints. Therefore, more investigations are required to assess the advantages and disadvantages of developing task-specific EXOs versus generalized EXOs for performing common tasks in construction workplaces.

Additionally, to protect the privacy of workers, regulations are needed regarding the collection and use of personal identifiable data collected from EXOs during their use by workers in construction workplaces.

#### 5.2.5. Mapping Solutions to Barriers

**Figure 3:**
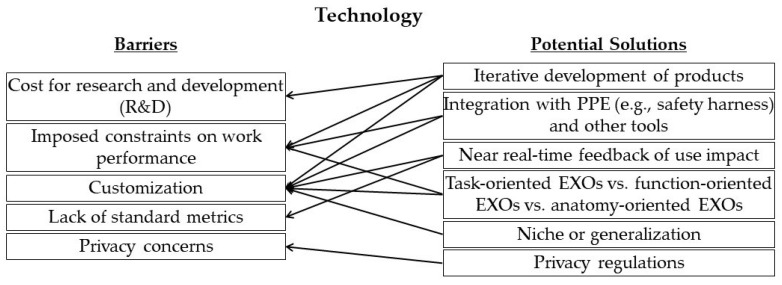
Mapping of potential solutions to identified barriers in the technology category.

The panel of experts discussed potential solutions for all the barriers in the technology category identified above. Figure 3 shows the mapping of barriers to potential solutions.

### 5.3. Organization

At the organizational level, the decision to adopt any technology is typically affected by factors such as technical–organizational match, organizational structure accommodation, management burden, and organizational culture. Therefore, perspectives from the organizational standpoint are important in exploring the adoption potential of EXOs in the construction industry.

#### 5.3.1. Results

Table 5 depicts the compiled outcomes from the discussion of the expert panel regarding the facilitators, barriers, and potential solutions in the organization category.

#### 5.3.2. Facilitators

Unions can play an influential role in driving the adoption of EXOs in the industry. Unions can promote the use of EXOs in their campaigns, meetings, and interactions with industry trades and thereby create greater awareness and appreciation of the potential benefits of using EXOs.

EXOs have the potential to reduce the risk of injuries among construction workers. If the number of injuries is reduced, insurance companies incur lower costs for worker compensation, thereby increasing profits for their businesses. Therefore, insurance companies can also be motivated to promote the adoption of EXOs by promoting these products to relevant business clients. Additionally, insurance companies can offer incentives, such as reduced insurance premiums, to construction companies that adopt EXOs at their jobsites.

#### 5.3.3. Barriers

At the organizational level, construction companies are required to make financial decisions about investments in EXOs based on factors such as benefits, costs, safety concerns, and staffing requirements at construction jobsites. There are also decisions to be made about the best uses of EXOs for construction tasks, which could lead to more administrative work for the organization. Moreover, if companies decide to use EXOs at their jobsites, they need to ensure that effective training programs are available to train workers in the proper use of EXOs. Additionally, construction companies could face increased liability from improper use of EXOs by workers, and such risks could discourage construction companies from adopting EXOs.

Construction companies follow standards and best practices with respect to using materials, tools, and equipment to achieve efficiency and quality of work at jobsites. When new technologies are introduced in a workplace, standards and best practices for the use of the technologies are needed to ensure that the project performance is maintained or improved. Unfortunately, such standards and best practices for EXO use in the construction industry have yet to be established, limiting organizational decision makers’ confidence regarding EXO adoption.

Corporate culture plays an important role in the successful adoption of new technologies. Many organizations become tied to performing their operations in an established way and are reluctant to change. The adoption of new technologies will result in a series of changes in areas such as work practices, policies, staffing, organizational structure, administration, and employee responsibilities. When a company’s culture is resistant to change, it can be exceedingly challenging to influence their beliefs, opinions, and attitudes about EXO use.

According to the Center for Construction Research and Training (CPWR), about 91% of US construction businesses have fewer than 20 employees [28]. For these small businesses with low annual revenues, the adoption of EXOs with high initial costs would be difficult.

#### 5.3.4. Potential Solutions to Barriers

Currently, EXOs are not commonly used on construction sites, and hence there are inadequate real-world data on EXO use in construction. Extant assessments of the impacts of EXOs are primarily based on experiments conducted in laboratory settings. To promote the adoption of EXOs in the construction industry, there is a need for more research studies performed at construction jobsites to gather data regarding the usability and performance of EXOs.

Systematic scientific studies can be supplemented by pilot implementation of EXOs to examine their feasibility in the construction industry. Evidence-based findings from such studies will reduce the knowledge gap regarding the applicability and benefits of EXO use in the construction industry and encourage companies to consider the adoption of EXOs.

If an accident occurs while using EXOs in construction workplaces, a standard procedure is necessary to analyze the event and determine the root causes for the accident, as well as the responsible parties, including the worker, the construction company, the EXO manufacturer, and the insurance company. Having an established process for addressing EXO-related accidents and injuries will help ensure that appropriate interventions are taken and that the impacted workers are adequately compensated.

Increased awareness and education in the construction industry regarding the benefits, potential, and proper use of EXOs can be impactful in driving the adoption of EXOs. Such education can be conducted at industry events, where EXO producers can demonstrate the products firsthand. In addition, reaching out to community leaders who may influence big corporations can help in the adoption process.

#### 5.3.5. Mapping Solutions to Barriers

**Figure 4:**
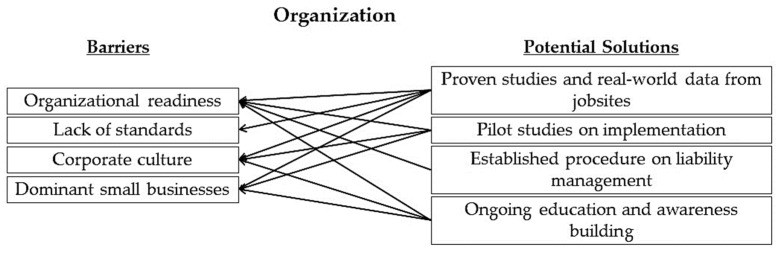
Mapping of potential solutions to identified barriers in the organization category.

Experts identified probable solutions to all the identified barriers in the organization category as indicated in Figure 4.

### 5.4. Policy/Regulation

While new technologies hold promise for improved performance, it is necessary to ensure that their implementation is responsible and will conduce to the prosperity of the industry in question. Policy attention is focused on the adverse effects of technological adoption, which is also necessary for decision making regarding the adoption of EXOs in the construction industry.

#### 5.4.1. Results

Table 6 presents the compiled outcomes from the discussion of the expert panel regarding the facilitators, barriers, and potential solutions in the policy/regulation category.

#### 5.4.2. Facilitators

Third-party organizations, such as unions, industry associations, and public agencies, can help initiate discussions on policies, standards, and regulation development for EXOs, which are vital to the adoption and implementation of EXOs in construction workplaces.

The US government’s Small Business Innovation Research (SBIR) and Small Business Technology Transfer (STTR) funding programs encourage domestic small businesses to engage in Federal Research/Research and Development (R/R&D) with the potential for commercialization [29]. The goal of these programs is to encourage technological innovation to meet the nation’s research and development needs and they can help drive EXO use by small businesses.

#### 5.4.3. Barriers

There are currently no established standards for EXOs, though some initiatives are underway, such as ASTM F48 on Exoskeletons and Exosuits [30], to provide guidelines for testing or assessing the performance and use of EXOs. EXOs are pieces of equipment that are susceptible to wear and tear. If an EXO breaks or malfunctions while a task is being performed, severe injuries and even fatalities can result. Therefore, regular inspections are essential to ensure that EXOs work properly. Any minor issue needs to be fixed immediately so that incidents can be avoided while workers are using the equipment at the jobsite. Regulations or policies are needed to govern or guide the maintenance of EXOs in a timely manner to ensure worker safety. Currently, such regulations or policies have not been established. Unions strive to ensure worker safety, and new or updated union rules may be needed to help trades implement this new technology at construction workplaces.

Relevant regulations made at the state/government level would accelerate EXO adoption. Nevertheless, the development of such regulations is difficult and painstakingly slow.

Differences in EXO-related policies across countries and regions present hurdles. Such differences may lead to confusion about the procedures and protocols for EXO use and pose challenges to EXO manufacturers and vendors with respect to standardizing their products.

Workers who use PPE while working in elevated conditions can face challenges in using EXOs. If workers need to use EXOs in elevated conditions in construction workplaces, it would be desirable to incorporate PPE with such EXOs.

This also raises the question of whether EXOs should be considered as PPE. The general duty clause states that all workers have a right to a safe and healthy workplace. Accordingly, there are specific regulations regarding PPE that all workers must follow on-site. If EXOs are considered PPE, then EXOs need to be regulated.

#### 5.4.4. Potential Solutions to Barriers

The availability of more scientific data will provide stronger evidence regarding EXOs’ benefits and risks. The data will assist in identifying opportunities for the improvement of EXOs and provide bases for regulation and policy development, which will drive the adoption of the technology in the construction industry.

Unions work relentlessly to protect the rights of workers. They can play a significant role in policy formulation by voicing demands to the authorities for the regulation of EXOs to ensure workers’ safety in construction workplaces. Other organizations, such as the Constructors Association of Western Pennsylvania (CAWP), can also play a vital role in this regard as they interact with Occupational Safety and Health Administration (OSHA) and can significantly influence policymaking.

To support EXO use by construction workers, training and education are essential for all associated roles, such as general contractors, project managers, superintendents, and trade workers. Training should include not only the proper use of EXOs but also all the policies and regulations (if any) regarding the safe use of EXOs and prevent any violation of regulations.

If there are businesses implementing EXOs in their workplaces, the dissemination of such endeavors may encourage other businesses to consider the use of EXOs. If their experience is satisfactory, these businesses can expand their use of EXOs and thereby encourage more businesses to adopt EXOs. A positive perception regarding EXOs may thereby grow within the industry, which will foster the formulation of relevant regulations and policies for EXO implementation.

The development of industry standards and best practices for EXOs by industrial associations as well as relevant agencies will encourage the adoption of EXOs.

The ANSI A10 is a series of American national standards published by the American Society of Safety Professionals (ASSP) that covers the safety requirements for activities related to construction and demolition operations. It provides safety regulations for every activity, from erecting scaffolding to handling explosives to pouring concrete. ANSI A10 drives OSHA regulations for construction. Therefore, it is plausible to develop a series of national standards for activities concerning the use of EXOs in the construction industry that ensure the safety of construction workers while using EXOs on jobsites.

There are strict regulations regarding work performed at elevated conditions by construction workers, such as wearing personal fall-arrest systems. Regulations on the use of EXOs at elevated conditions will aid their adoption.

#### 5.4.5. Mapping Solutions to Barriers

**Figure 5:**
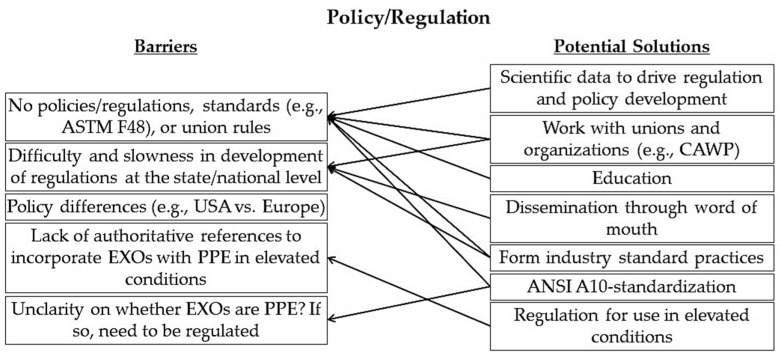
Mapping of potential solutions to identified barriers in the policy/regulation category.

Figure 5 shows the mapping of potential solutions to the barriers identified in the policy/regulation category by the expert panel. The barrier “Policy differences (e.g., US vs. Europe)” requires further investigation as no feasible solution was immediately evident from the workshop discussion.

### 5.5. Ergonomics/Safety

EXOs are wearable technologies that have direct contact with the human body. The protection provided as well as potential ergonomic injury risks and safety hazards induced by the use of EXOs during work will be a decisive factor in the adoption of EXO technology in workplaces in the construction industry.

#### 5.5.1. Results

The outcomes from the expert panel’s discussion regarding facilitators, barriers, and potential solutions in the ergonomics/safety category are compiled in Table 7.

#### 5.5.2. Facilitators

A significant number of workers in the construction industry suffer from musculoskeletal disorders (MSDs) [31]. Construction companies also incur immense losses due to days away from work and compensation payments to impacted workers. EXOs have the potential to reduce the risks of MSDs among workers by providing additional means of protection for workers performing physically demanding tasks. Such benefits can encourage both workers and employers to adopt EXOs in construction workplaces.

#### 5.5.3. Barriers

There are several safety concerns regarding the use of EXOs. First, there are no safety inspection protocols that exist for EXOs to ensure the safety of using the devices. If a device fails while performing a task, there may be severe safety consequences. For example, suppose a worker is carrying a load that exceeds his physical capacity and the EXO fails while the load is being held overhead or over the shoulder. In such a case, the total load may fall and cause severe injuries to the worker. Second, active EXOs are powered by external sources that give a worker strength significantly greater than his own physical strength. When a worker is constantly performing heavy-duty work while wearing an EXO, s/he might develop a misconception regarding his/her capability. This misconception about personal augmented capability might lead to the worker intuitively performing heavy work when not wearing an EXO, which could result in injury. Third, passive EXOs use other body muscles to counterbalance forces by the redistribution of loads. This may cause residual injuries to other body parts. Fourth, due to continuous support from EXOs, the supported parts of the body might become weaker, leading to muscle atrophy. Finally, workers may have pre-existing conditions that may deteriorate due to the use of EXOs. There are currently no methods to definitively ascertain that EXOs would not negatively affect workers under these conditions.

There are several additional safety hazards associated with EXOs. For example, active EXOs are typically heavy and not very flexible for movement. Therefore, while working in an elevated condition, a worker might lose their balance and fall, sustaining severe injuries. In addition, different weather conditions, such as rain or snow, can cause accidents due to slipping, as movement with EXOs can be unnatural. Many active EXOs are powered by electric sources and need to be connected to a source with wires. There is a possibility of electrocution of the worker while the EXO is connected to the source, and the wires may get tangled with the worker and result in an accident.

EXOs need to be comfortable for workers to wear as they may spend long periods of time during shifts in these suits. If EXOs are not comfortable, there might be a risk of MSDs, and this would defeat the whole purpose of using EXOs. Moreover, suits need to be tailor-made for each worker so that the mechanism can work perfectly. Otherwise, pressure might be exerted on unintended body parts, thereby increasing the risk of MSDs. Heavy and bulky EXOs together with PPE on construction sites are harder to carry and place constraints on movement for workers. If a worker wears an EXO for many hours, as with back belts, over-exertion of the muscles being used might result. Furthermore, wearing EXOs for long durations can cause pain, soreness, and discomfort for workers.

For EXOs to be adopted in the construction industry, objective measurements related to ergonomics and safety in terms of benefits and risks are necessary. However, reliable methods for and research in the literature on performing such measurements are currently unavailable. EXOs are new to the construction industry, and studies with EXOs have primarily been conducted in laboratory settings; hence, there are still many unknowns regarding the use of this technology in real-world construction tasks. These knowledge gaps also hinder the development of confidence in the industry regarding the use of EXOs.

#### 5.5.4. Potential Solutions to Barriers

Most of the EXOs in use are task-oriented. Ensuring the proper selection of EXO products for specific tasks is therefore important. If an EXO is used to perform tasks for which it was not designed, it might exert undesired pressure on body parts, increasing the risk of MSDs.

If real-life case studies of EXO use can be conducted with construction workers at actual jobsites, then the performance data generated from these studies will provide research-based evidence to encourage the adoption of EXOs. These data can also facilitate further improvement of EXOs to better satisfy the actual task and workplace requirements and constraints.

Inspection protocols are essential to ensure the safety of EXO devices and prevent any accidents while performing tasks with EXOs. A specific inspection and maintenance schedule might be desirable to identify wear and tear and malfunctions in a timely manner and perform necessary repair and replacement activities so as to ensure EXO safety. Policies or regulations might be needed to enforce implementation of the protocols so that all organizations will be obliged to follow them.

Muscle atrophy caused by continual use of EXOs can be minimized by establishing best practices regarding the safe use of EXOs. Training programs for such best practices for those who wear EXOs in construction workplaces can be beneficial in terms of reducing muscle atrophy.

The utilization of EXOs at construction jobsites has great potential to reduce MSDs and increase productivity. More examples are needed as evidence and encouragements for prospective users to adopt EXOs, even if the results are not always positive. Negative results (if obtained) can also be valuable in helping EXO manufacturers improve their products, ensuring that future products are safer for use in construction workplaces.

Regulations are required to ensure safety while using EXOs. Safety guidelines are essential, and supervisors must ensure that the guidelines are followed by workers so that the risks of MSDs or injuries due to improper use of EXOs are minimized.

#### 5.5.5. Mapping Solutions to Barriers

**Figure 6:**
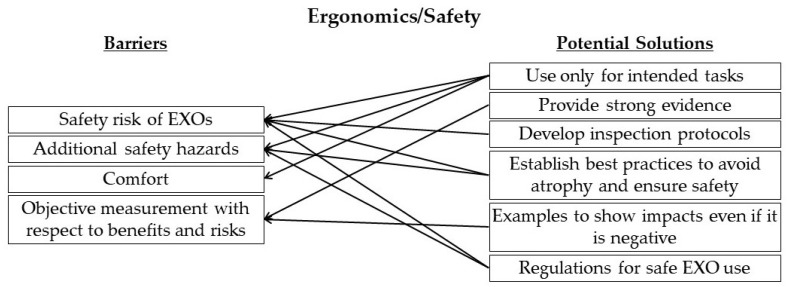
Mapping of potential solutions to identified barriers in the ergonomics/safety category.

Figure 6 portrays the mapping of potential solutions to the barriers identified in the ergonomics/safety category by the expert panel during the workshop.

### 5.6. End Users (Trade Professionals)

The success of any technology adoption hinges on end users’ (trade professionals in this case) acceptance of and confidence in utilizing the technology in question. Understanding trade professionals’ readiness components, such as skill learning requirements, physical and psychological safety, and perceivable value, is critical to the adoption of EXOs in construction.

#### 5.6.1. Results

The outcomes regarding the facilitators, barriers, and potential solutions in the end users (trade professionals) category resulting from the expert panel discussion are compiled in Table 8.

#### 5.6.2. Facilitators

Construction workers are required to perform physically demanding work that is impractical for aged workers and these people, consequently, are often forced to leave the industry. EXOs offer the potential to help aged workers perform heavy-duty work by providing additional support and protection to relevant body parts and thereby enabling this population to continue their employment for an extended period of time. The benefits of retaining aged workers through the use of EXOs may be an impetus for the adoption of EXOs in the construction industry.

Building the self-efficacy of workers through the use of EXOs may facilitate their adoption. Self-efficacy refers to an individual’s belief in their capacity to act in the ways necessary to achieve specific goals. If workers have hands-on experience with EXOs, they can develop a greater appreciation of the benefits of EXOs, such as augmented capacity, reduced pressure on vulnerable body parts, as well as reduced exhaustion or fatigue over time. Such self-efficacy can motivate workers to adopt EXOs and increase productivity while reducing the risk of MSDs.

Enhancing the ease of use of EXOs would facilitate their adoption. Workers often have concerns regarding complex products and hence are unwilling to try them, which impedes the adoption process. If EXOs can be made easy to use for workers, they would be more likely to be willing to try EXOs in construction work and drive adoption.

Reduced costs are an important factor in the adoption of EXO products. Affordable EXOs will make it possible for independent workers and small contractors to procure the products and use them for their businesses. General contractors and large corporates would also benefit from lower-priced EXOs for the large-scale deployment of EXOs in the industry.

It would be beneficial if the worker population had a positive perception of EXOs so that they could encourage union leaders, industry associations, and employers to adopt EXOs.

Tangible results in terms of physical benefits and productivity would be strong evidence to encourage workers to try EXOs. If such tangible results can be presented to workers, showing that they can benefit from the use of EXOs in terms of improved safety, enhanced capabilities, or increased productivity, workers are likely to be motivated to adopt EXOs at jobsites.

Training can provide workers with firsthand experience about the proper use of EXOs, allowing them to formulate personal opinions based on their own experience, which might encourage them to adopt EXOs.

Manufacturers can play a significant role in the adoption process of EXOs in the construction industry through activities such as demonstrations at exhibitions, providing training in apprentice programs and trade schools, the publication of short manuals tailored to the industry, and provision of technical support to contractors, all of which would increase the opportunities for worker–EXO interactions within the industry and thereby facilitate their adoption. Manufacturers can also help in developing case studies by collaborating with the construction industry and the academic research community, which, in turn, will increase their products’ visibility and encourage adoption.

#### 5.6.3. Barriers

From the perspective of end users (workers), EXOs currently are considered difficult to use and becoming proficient with EXOs involves a steep learning curve. This barrier can discourage trade professionals from attempting to use these products and therefore hinder EXO adoption in the construction industry.

In the course of their work experience, workers will have cultivated their preferred methods and habits for routine daily jobs and hence will be reluctant and resistant to change and to trying out new technologies.

EXOs can be equipped with sensors to collect real-time or asynchronous data for performance monitoring and safety inspection. However, this can raise a concern among the end users (i.e., the workers) that they will be tracked and lose their privacy. Such concerns can pose barriers to the use and adoption of EXOs by end users.

Workers’ satisfaction plays a vital role in the adoption of EXOs, as they are the end users of the products. Nevertheless, in practice, workers are often not fully consulted in the process of adoption and implementation. For this reason, workers may not be well informed and thereby have misperceptions regarding the effects, functions, benefits, and potential risks of using EXOs. As such, workers may be less enthusiastic about using EXOs. Workers need to develop a personal appreciation of the benefits of using EXOS in order to facilitate their adoption in the workplace.

EXOs are designed to make workers more efficient and productive. However, this raises the concern that the increased productivity with EXOs could translate into reduced need for labor. Workers may therefore perceive the adoption of EXOs in the industry as increasing the risk of their unemployment. In addition, there is a concern about workers’ rights, i.e., whether workers have the right to refuse to use EXOs if they do not want to. Additionally, some workers may have chronic health issues that prevent them from wearing EXOs. If a company requires its workers to use EXOs, then those who are unwilling or unable to wear EXOs may be concerned about their employment.

As construction devices or equipment, EXOs are subject to wear and tear, deterioration, and unexpected breakdown. As workers will rely on the functional support of EXOs while they are wearing them in work, unexpected malfunctions of EXOs during usage could potentially cause harm. As a result, workers may be concerned about the injuries that may result from EXO failure while in use.

Workers may disapprove of new technologies due to potential stigma associated with the use of EXOs. Workers may fear that construction automation may make them redundant and result in unemployment.

Active EXOs are usually bulky in size and require a power source that sometimes requires an electrical connection. This requirement can cause incidents, such as entanglement with connecting wires (if needed). Furthermore, for certain types of EXOs, working near electromagnetic fields can be risky.

#### 5.6.4. Potential Solutions to Barriers

Providing hands-on experience with easy-to-use EXOs (including easy donning and doffing, adjustment, and operation) will promote their adoption among workers. Such experiences will help workers build their own unbiased perceptions about the usability and comfort of the devices that will facilitate the acceptance and adoption of EXOs among the worker population.

As many unknowns exist regarding the use of EXOs in construction settings, continued research is essential for promoting the adoption of this technology in the construction industry. Education and training of different sorts at various stages are also necessary to increase awareness and use of the technology among current and future workers. Greater awareness regarding the benefits of EXOs may also trigger workers’ interests in seeking education and training. Proper training would allow for more interactions of the workers with EXOs and provide them with the needed skills for the use of EXOs, both of which would be positive for the adoption of EXOs in the construction industry.

Leadership from both employers and unions can play an important role in the adoption process of EXOs through influence on and the sharing of values with their employees, members, and followers, among whom many are workers. Workers could be further informed about the potential benefits of EXOs through campaigns, through which they may acquire more information, opinions, experience, and therefore ideas about possible EXO use in construction.

As the end users, workers’ satisfaction is a key factor in the acceptance of any new technology in workplaces. Worker feedback needs to be sought and incorporated while designing EXOs intended for use in construction. Bidirectional communications would benefit both the workers (end users) and the EXO manufacturers. Growing satisfaction with EXOs amongst workers will drive the adoption of this technology in construction.

The visual appearance and appeal of new technologies can also affect their adoption by users. Construction workers wear different items of safety gear to perform their tasks. If EXOs can be designed in such a way that their appearance seems “invisible” (i.e., not noticeable) or appealing (e.g., fashionable or like superhero outfits), they might be attractive for workers, especially future young workers, and encourage adoption at jobsites.

Identifying ways to generate, collect, and disseminate perceivable and measurable benefits of EXOs so that workers can comprehend and appreciate them is vital to the adoption process. This may be achieved through case studies and examples of applications and dissemination of the benefits through campaigns, visual presentations, training programs, workshops, and word of mouth.

#### 5.6.5. Mapping Solutions to Barriers

**Figure 7:**
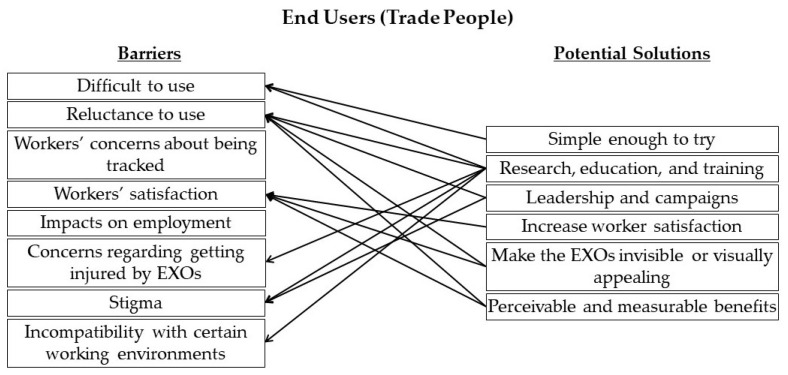
Mapping of potential solutions to identified barriers in the end users (trade professionals) category.

Figure 7 shows the mapping of potential solutions to the barriers identified in the end users (trade professionals) category discussed by the expert panel. The barrier “Impacts on employment” requires further investigation before potential solutions can be proposed.

### 5.7. Top Barriers to EXO Adoption

The expert panel reached a consensus on the top barriers to EXO adoption, which are listed in Table 9 with their associated categories. These barriers and their potential solutions have been discussed in the prior subsections. The panel particularly emphasized the importance of education, including educating contractors, workers, engineers, and even future generations of engineers and workers. Human-centric design and development of EXOs is another factor prioritized by the panel as being critical for the industry’s adoption of the technology.

## 6. Discussion

EXOs are a new technology for the construction industry with the potential to improve the industry by reducing WMSDs and improving productivity. Several research works have highlighted the potential benefits of using EXOs in construction workplaces, but the adoption of EXOs in the industry has been very limited. This study attempted to identify the reasons underlying the limited utilization of EXOs in construction by gaining inputs from and establishing a consensus among a panel of experts representing academia, industry, and a government agency through a Delphi process to obtain a holistic understanding of the facilitators and barriers and their potential solutions. Although the experts involved in this study were all from the US, the outcomes still shed light on the development of EXOs for construction adoption and utilization in many other countries and regions, considering that the nature of construction tasks and work settings are alike worldwide, the lens through which the potential for EXO adoption has been viewed, and the positioning of the present study based on the state of research in assessments of EXO use in construction. The present study determined that a wide range of issues remain to be addressed for using EXOs in construction workplaces, and the implications for different stakeholders are presented below.

### 6.1. Implications for the Academic/Research Community

Researchers have been performing experiments and analyses on EXOs to gain insights into the potential uses of EXOs in construction and their benefits. This study provides the research community with additional information to guide future efforts. The complete list of barriers with the prioritized ones identified by the experts for EXO use in real-life construction settings may set the stage for future research efforts to find solutions to solve the barriers and thereby facilitate the adoption process.

### 6.2. Implications for Government and Public Agencies

The adoption of EXOs in construction workplaces will require new policies and regulations to ensure the safety of workers while wearing EXOs and support them if injuries or accidents occur due to EXO use. Specific policies are also required to determine which party is liable for training arrangements and possible injuries (if any) due to continuous EXO use. The findings regarding desirable policies and regulations discussed earlier in this paper can aid the appropriate government and public agencies in the formulation of policies and regulations for EXO users, businesses, and manufacturers.

### 6.3. Implications for EXO Manufacturers

EXOs are rapidly evolving technologies. While EXO usage is more prevalent in several other sectors, the technology needs further development and modifications/improvements to adapt it to construction applications. This study has identified several critical requirements that highlight the modifications necessary for EXOs to be used in construction workplaces. For example, this study determined that EXOs need to be integrated with PPE to work in construction environments. It also emphasized that EXOs need to be lightweight and tailor-made for ease of use by construction workers. The findings and recommendations from this study can help EXO manufacturers in improving EXOs to meet the requirements of the construction industry. The availability of EXOs that are well-suited to construction tasks will encourage construction firms to invest in them, thereby benefiting both EXO manufacturers and the construction industry overall.

### 6.4. Implications for Construction Firms

At present, most construction businesses and organizations remain unconvinced about the benefits of EXO use in construction workplaces in terms of costs, safety, and efficacy. While several studies have been conducted to assess the usability of extant EXOs in construction, feasibility studies and real-world case studies of EXO applications in construction are still required to influence organizations toward the adoption of this new technology. This study presents a comprehensive view of the barriers that construction organizations are facing and also identifies key factors that can encourage businesses to adopt EXOs.

### 6.5. Implications for Trade Professionals (End Users)

The wide adoption of EXOs in the construction industry will strongly depend on their acceptance by trade professionals (end users). The variety of barriers that can cause rejection of EXO products among workers or slow down the process of EXO adoption in construction workplaces has been identified in this study. The potential solutions to these barriers have been discussed. The identification of the barriers will help inform end users regarding the potential risks given the status quo regarding EXO products and enable the worker community to understand the steps needed for the adoption of EXOs to be successful. Some of the technological modifications required to meet the needs in construction environments have also been identified from the end users’ perspective. Manufacturers and researchers are encouraged to leverage these findings to continually improve EXOs in collaboration with workers.

### 6.6. Implications for Other Industries

Many other industries, such as manufacturing [32], agriculture [33,34], baggage handling [35], logistics [36,37], medical care [38], and the automotive [39,40,41] and ship maintenance industries [42], are exploring potential uses for EXOs. Currently, the rate of EXO use in these industries is also limited. The results of this study may shed light on the pros, cons, opportunities, and challenges of EXO development with respect to adoption and use in these industries. For instance, the authors believe that the facilitators, barriers, and potential solutions to the barriers identified for the business and organizations categories in this paper are likely to be relevant to other industries. While considering technological modifications of EXO products necessary for a certain industry, the outcomes in the technology category might provide useful insights regarding the factors that need to be scrutinized. The outcomes with respect to policies and regulations from this paper may also be thought-provoking for other industries, as worker safety concerns are quite similar across industries. Regarding the ergonomics/safety category, the safety concerns elaborated on in this paper that need to be addressed regarding the adoption of EXOs are pertinent to many of the workers in other industries, as they, too, are susceptible to WMSDs. Finally, the workers’ concerns identified in the end user category represent the concerns of workers, regardless of the industries they belong to. As a result, the findings of this paper are beneficial not only for construction but also many other industries that are actively evaluating the potential use of EXOs and working toward their adoption.

## 7. Conclusions

Using a three-phase Delphi approach, this study identified facilitators, barriers, and potential solutions regarding the adoption of EXOs in the construction industry, considering the substantial benefits that this new technology can potentially offer. A panel of experts from academia, industry, and government with relevant knowledge and experience gathered in a workshop to provide inputs, engage in discussion, and reach a consensus. The outcomes will help provide a better understanding of the benefits, risks, and opportunities of EXO use in the construction industry, while shedding light on ongoing developments and endeavors needed to study the envisioned future of technology, workers, and work in construction.

## Figures and Tables

**Table 1:** Composition of the expert panel.

Academia	Industry	Government Agency
Construction × 2	Contractors × 2	NIOSH * × 5
Mechanics × 2	EXO Manufacturers × 2	
Robotics × 1	Risk Consultancy and Insurance × 1	
Occupational Safety and Health × 1		
Psychology × 1		
Economics × 1		

* NIOSH = National Institute for Occupational Safety and Health. Five experts participated from NIOSH, representing three different relevant divisions.

**Table 2:** Demographic information and percentage composition of the panel.

Years of Work Experience		Composition of the Expert Panel	
0–5 years	2 (11.1%)	Academia	8 (44.4%)
6–10 years	4 (22.2%)	Industry	5 (27.8%)
11–20 years	6 (33.3%)	Government Agency	5 (27.8%)
21–30 years	5 (27.8%)		
>30 years	1 (5.6%)		

**Table 3:** Summary of business category outcomes.

	Business
Facilitators	Increased efficiency (morale and productivity) Potential economic impacts ○ Create job opportunities for individuals with expertise in EXO use, training, and certification ○ Reduce injuries and worker compensation costs ○ Alleviate labor shortages
Barriers	Cost of EXO ownership ○ Initial purchase costs and ongoing maintenance costs ○ Owned by worker or by company? Lack of belief in claimed benefits ○ Not enough objective scientific/case study data to validate EXO manufacturer claims Return on investment (ROI) quantification ○ Difficult to determine benefits associated with EXO-enabled work practices ○ How to measure/quantify EXO impact on injury prevention and the corresponding economic impact on businesses Staffing and implementation ○ Too busy to trial ○ Lack of professionals to train workers on efficient use of EXOs ○ Lack of management of EXO inventory and allocation to workers as well as maintenance of EXOs ○ Lack of experts for implementation (certification, education, and healthcare) Inadequate EXO product options Unknown impacts on work-pattern shift
Potential Solutions to Barriers	Work with unions, trade schools, contractors, associations, and apprentice training programs Provide training to workers ○ Increase awareness among construction stakeholders ○ Provide opportunities to use and conduct trials with EXOs outside workplaces More general education in high school and higher education Doctors’ recommendations Work with EXO experts More scientific data to validate EXO benefits More real-world case studies

**Table 4:** Summary of technology category outcomes.

	Technology
Facilitators	Technology investment sector Real-time feedback technologies
Barriers	Cost for research and development (R&D) ○ Risk of investment Imposed constraints on work performance ○ Bulky size ○ Donning and doffing ○ Motion constraints Customization ○ Compatibility and interaction with existing personal protective equipment (PPE) (e.g., safety harness) ○ Mostly task-specific ○ Active vs. passive EXOs Lack of standard metrics ○ Testing procedure differences Privacy concerns
Potential Solutions to Barriers	Iterative development of products ○ Improved time of adjustment ○ Ease of immediate use ○ Lighter devices Integration with PPE (e.g., safety harness) and other tools Near real-time feedback of use impact Task-oriented EXOs vs. function-oriented EXOs vs. anatomy-oriented EXOs Niche or generalization Privacy regulations

**Table 5:** Summary of organization category outcomes.

	Organization
Facilitators	Unions Insurance industry
Barriers	Organizational readiness ○ Financial decisions ○ Use of technology ○ More administrative work ○ Additional training arrangements ○ Liabilities Lack of standards Corporate culture Dominant small businesses
Potential Solutions to Barriers	Proven studies and real-world data from jobsites Pilot studies on implementation Established procedure on liability management Ongoing education and awareness building

**Table 6:** Summary of policy/regulation category outcomes.

	Policy/Regulation
Facilitators	Third-party promotion SBIR and STTR programs
Barriers	No policies/regulations, standards (e.g., ASTM F48), or union rules Difficulty and slowness in development of regulations at the state/national level Policy differences (e.g., US vs. Europe) Lack of authoritative references to incorporate EXOs with PPE in elevated conditions Unclarity about whether EXOs are PPE? If so, there will be a need for regulation
Potential Solutions to Barriers	Scientific data to drive regulation and policy development Work with unions and organizations (e.g., CAWP) Education Dissemination through word of mouth Form industry standard practices ANSI A10-standardization Regulation for use in elevated conditions

SBIR: Small Business Innovation Research; STTR: Small Business Technology Transfer; ASTM: American Society for Testing and Materials; CAWP: Constructors Association of Western PA; ANSI: American National Standards Institute; PPE: personal protective equipment.

**Table 7:** Summary of ergonomics/safety category outcomes.

	Ergonomics/Safety
Facilitators	Prevention of musculoskeletal disorders (MSDs)
Barriers	Safety risk of EXOs ○ Device failure ○ Misconception about the augmented capability ○ Residual injury risks ○ Muscle atrophy ○ Pre-existing conditions/injuries Additional safety hazards ○ Loss of balance ○ Different weather conditions ○ Different work conditions Comfort ○ Movement restraints ○ Device weight ○ Over-exertion, as with back belts ○ Pain, soreness, and discomfort Objective measurements with respect to benefits and risks ○ No reliable methods or literature ○ Still many unknowns in real-life use
Potential Solutions to Barriers	Use only for intended tasks Provide strong evidence Develop inspection protocols Establish best practices to avoid atrophy and ensure safety Examples to show impacts, even if negative Regulations for safe EXO use

**Table 8:** Summary of end users (trade professionals) category outcomes.

	End Users (Trade Professionals)
Facilitators	Retention of aged workers Self-efficacy of workers Increased ease of use Reduced costs Positive product perception Tangible results (physical and productivity-related) Training Support from EXO manufacturers
Barriers	Difficult to use ○ Steep learning curve Reluctance to use Workers’ concerns about being tracked Workers’ satisfaction ○ Workers not consulted ○ Perception about tangible benefits (e.g., percentage reduction in injuries) Impacts on employment ○ Does this impact employment number? ○ Do workers have the right to refuse? ○ Does this impact workers’ ability to maintain employment based on health/ability to use? Concerns regarding injuries due to EXOs ○ Will equipment failure result in injuries? Stigma Incompatibility with certain working environments
Potential Solutions to Barriers	Simple enough to try Research, education, and training Leadership and campaigns Increase worker satisfaction Make the EXOs invisible or visually appealing Perceivable and measurable benefits

**Table 9:** Top barriers identified by the expert panel with associated categories.

Top Barriers	Associated Categories
Lack of education	Business, Organization,
	Policy/regulation,
	End Users (Trade Professionals)
Lack of worker engagement from the beginning (e.g., trade school)	End Users (Trade Professionals)
Lack of trust in the devices	Business
Lack of subjective user experience (short-term vs. long-term)	Business, Organization,
	Ergonomics/Safety,
	End Users (Trade Professionals)
Lack of comprehensive evaluations (worker responses, short-term vs. long-term, long way to validation)	Business, Organization,
	Ergonomics/Safety,
	End Users (Trade Professionals)
Limited applications	Business, Technology,
	Ergonomics/Safety,
	End Users (Trade Professionals)
Lack of easy access to EXOs (including quick trials, onsite demonstrations, and resources for troubleshooting)	Business, End Users (Trade Professionals)
Few devices designed for construction work (need to wear multiple devices at the same time, as they are not integrated with PPE)	Business, Technology,
	Policy/Regulation

## Data Availability

Not applicable.
